# Golden insights from a silver superatom

**DOI:** 10.1093/nsr/nwae267

**Published:** 2024-07-30

**Authors:** Zhucheng Yang, Jianping Xie

**Affiliations:** Joint School of National University of Singapore and Tianjin University, International Campus of Tianjin University, China; Department of Chemical and Biomolecular Engineering, National University of Singapore, Singapore; Joint School of National University of Singapore and Tianjin University, International Campus of Tianjin University, China; Department of Chemical and Biomolecular Engineering, National University of Singapore, Singapore

Silver and gold, both members of the coinage group, have contrasting properties despite their similar atomic sizes and structures. Historically, humanity has been captivated by these metals’ unique attributes. In past decades, scientists have delved into their fundamental differences at the nanoscale, where atomically precise Ag and Au clusters play significant roles. Though ‘golden’ insights into these elemental disparities would be enabled by comparable Ag and Au clusters, progress with regard to the synthesis of these has not been in concert. While the first monodisperse Au_25_(SR)_18_ was reported in 2005 [1], its silver analogue, Ag_25_(SR)_18_, was not reported until 2015 [2]. This delay highlights the significant challenges in synthesis associated with the more reactive silver clusters. Now, Zhan Zhou, Lu-Fang Ma, Shuang-Quan Zang and their team have filled a significant gap by synthesizing an unprecedented Ag_13_ cluster, advancing the study of icosahedral superatomic clusters and offering ‘golden’ insights into cluster analogue chemistry [3].

In their study, the team synthesized a pair of analogue clusters (Au_13_ and Ag_13_) and a series of Ag*_n_*Au_13-_*_n_* clusters via doping (Fig. 1). This synthesis and doping approach removes the impact of motifs on electronic states, and also features identical protecting ligands and structures. This allows for a comprehensive comparative study on optical properties and electron dynamics to reveal the element-dependent emission. Despite having similar structures and the same 1S^2^|1P^6^ closed-shell electron configuration, the Ag_13_ solution exhibited no luminescence, while the Au_13_ solution displayed bright near-infrared (NIR) luminescence with a quantum yield of up to 45%. The density functional theory calculation and femtosecond-nanosecond transient absorption both support the theory that the evolution from Ag_13_ to Au_13_ leads to a change in the clusters’ energy level and structural rigidity with the increase in gold atom content. This doping alters the excited-state electronic structure, and the electron transfer process changes from local excitation (LE) to charge transfer to LE. This work not only enriches the superatom family, but also provides ‘golden’ insights into the evolution of superatoms’ optical properties. Moreover, the NIR-emissive Au_13_ was chemically modified with a hydrophilic shell to enhance its biocompatibility, demonstrating its potential in bio-imaging.

**Figure 1.**
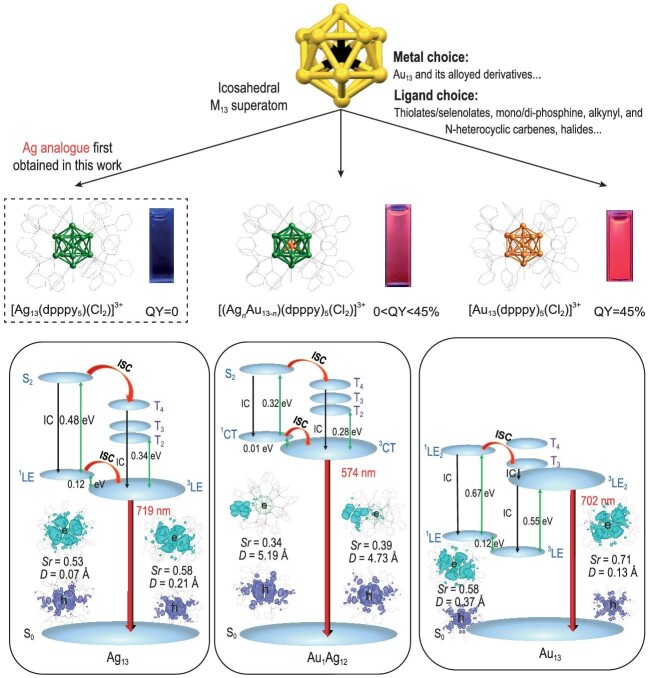
Schematic illustrations of the M_13_ products, their emission properties and energy diagrams. (Left) Ag_13_, (middle) Au_1_Ag_12_, (right) Au_13_. The top panel briefly summarizes the family of icosahedral superatomic clusters. Adapted from ref. [3].

In the context of nanochemistry development and synthetic progress in recent decades, the M_13_ cluster stands out as a promising model of superatoms with versatile alloyed compositions and diverse protecting ligands (e.g. thiolates, di-phosphine, alkynyl and N-heterocyclic carbenes) [4]. This family, now including Ag_13_, removes the impact of M(I) motifs in conventional clusters, allowing any property changes to be attributed to subtle composition differences [5]. We envision that this enriched M_13_ family, combined with electron dynamics studies, will provide more insight into the fundamentals and applications of superatoms. Additionally, the pursuit of biocompatibility for further biomedical applications of icosahedral superatomic clusters indicates the need for methodological innovations to directly obtain water-soluble ligand-protected counterparts.

In summary, the Ag_13_ superatomic cluster synthesized in this work enriches the superatom family. From this silver analogue, the authors provide ‘golden’ insights into atomic-level relations between core compositions and the mechanism of the phosphorescence. Future efforts to diversify this family, including water-soluble counterparts, and to precisely elucidate structure–property relationships, are anticipated to advance the field.

## References

[1] Negishi Y, Nobusada K, Tsukuda T. J Am Chem Soc 2005; 127: 5261–70. 10.1021/ja042218h 15810862

[2] Joshi CP, Bootharaju MS, Alhilaly MJ et al. J Am Chem Soc 2015; 137: 11578–81. 10.1021/jacs.5b07088 26322865

[3] He WM, Hu JH, Cui YJ et al. Natl Sci Rev 2024; 11: nwae174. 10.1093/nsr/nwae174 38887544 PMC11182670

[4] Narouz MR, Takano S, Lummis PA et al. J Am Chem Soc 2019; 141: 14997–5002. 10.1021/jacs.9b07854 31497943

[5] Hirai H, Takano S, Nakashima T et al. Angew Chem Int Ed 2022; 61: e202207290. 10.1002/anie.202207290 35608869

